# Second site escape of a T20-dependent HIV-1 variant by a single amino acid change in the CD4 binding region of the envelope glycoprotein

**DOI:** 10.1186/1742-4690-3-84

**Published:** 2006-11-29

**Authors:** Chris E Baldwin, Ben Berkhout

**Affiliations:** 1 Laboratory of Experimental Virology, Department of Medical Microbiology, Center for Infection and Immunity Amsterdam (CINIMA), Academic Medical Center of the University of Amsterdam, The Netherlands

## Abstract

**Background:**

We previously described the selection of a T20-dependent human immunodeficiency virus type-1 (HIV-1) variant in a patient on T20 therapy. The fusion inhibitor T20 targets the viral envelope (Env) protein by blocking a conformational switch that is critical for viral entry into the host cell. T20-dependent viral entry is the result of 2 mutations in Env (GIA-SKY), creating a protein that undergoes a premature conformational switch, and the presence of T20 prevents this premature switch and rescues viral entry. In the present study, we performed 6 independent evolution experiments with the T20-dependent HIV-1 variant in the absence of T20, with the aim to identify second site compensatory changes, which may provide new mechanistic insights into Env function and the T20-dependence mechanism.

**Results:**

Escape variants with improved replication capacity appeared within 42 days in 5 evolution cultures. Strikingly, 3 cultures revealed the same single amino acid change in the CD4 binding region of Env (glycine at position 431 substituted for arginine: G431R). This mutation was sufficient to abolish the T20-dependence phenotype and restore viral replication in the absence of T20. The GIA-SKY-G431R escape variant produces an Env protein that exhibits reduced syncytia formation and reduced cell-cell fusion activity. The escape variant was more sensitive to an antibody acting on an early gp41 intermediate, suggesting that the G431R mutation helps preserve a pre-fusion Env conformation, similar to T20 action. The escape variant was also less sensitive to soluble CD4, suggesting a reduced CD4 receptor affinity.

**Conclusion:**

The forced evolution experiments indicate that the premature conformational switch of the T20-dependent HIV-1 Env variant (GIA-SKY) can be corrected by a second site mutation in Env (GIA-SKY-G431R) that affects the interaction with the CD4 receptor.

## Background

Host cell entry of Human Immunodeficiency Virus type-1 (HIV-1) is a critical step in the virus life cycle, which requires the recognition of the host cell receptor CD4 and a co-receptor, CCR5 or CXCR4, by the viral envelope (Env) glycoprotein. Env is arranged on the virus particle as trimeric spikes, comprising three gp120 and three gp41 molecules, anchored within the viral membrane via the gp41 transmembrane (TM) domain. Binding of the surface subunit gp120 to CD4 and a co-receptor on the T-cell surface triggers conformational changes in the Env complex, leading to the insertion of the hydrophobic N-terminal fusion peptide (FP) of gp41 into the target cell membrane (reviewed in [1]). Subsequent changes within the gp41 ectodomain (gp41e) involve two leucine zipper-like motifs; heptad repeat 1 (HR1) and heptad repeat 2 (HR2). Ultimately, HR1 and HR2 from three gp41 molecules assemble into a highly stable 6-helix bundle structure, which juxtaposes the viral and cellular membranes for the fusion event [2-4]. The change in free energy associated with this structural transition within gp41e is predicted to be sufficient to cause lipid mixing and membrane fusion [5,6]. Peptide fusion inhibitors that bind to one of the HR motifs can block this conformational switch, and thus inhibit viral entry [7-10].

The fusion inhibitor T20 (also called DP-178, Enfuvirtide and Fuzeon™) is the most clinically advanced drug of a new class of antivirals designed to inhibit viral entry [11]. T20 is a synthetic 36 amino acid peptide derived from the C-terminal region of HR2 [8,12]. By competitive binding to HR1, T20 blocks the formation of the 6-helix bundle, which is a prerequisite for membrane fusion and viral entry [8,13]. T20 has also been proposed to have additional target sites within Env; the C4 region of gp120 and the viral membrane proximal region of gp41e [14-18]. The C4 region in gp120 is involved in CD4 and co-receptor engagement and differences in how Env engages its receptors can influence T20 sensitivity [14,15].

HIV-1 variants that are resistant to this compound have been described and resistance mutations have been identified within the viral quasispecies of patients on T20 therapy [19-24]. Sequence analysis of the resistant viral population revealed the acquisition of mutations mainly within a stretch of three HR1 amino acids, glycine-isoleucine-valine (further referred to as the GIV sequence, HXB2 amino acid positions 547 to 549 of gp160). In addition, mutations flanking this region (amino acids 550–556 of HR1) have also been proposed to confer a distinct level of resistance to T20 [25-27].

Recently, we performed a genetic analysis of the entire HIV-1 gp41e of the viral population from a patient that failed on T20 therapy [20]. Sequence analysis revealed the acquisition of the T20-resistance mutation GIA (GIV to GIA; mutated amino acid underlined) in HR1. We also documented a subsequent change in the three amino acid SNY sequence of the HR2 domain (SNY to SKY). We demonstrated that the HR1–HR2 double mutant (GIA-SKY), which dominated the viral population after 32 weeks of therapy, was not only highly resistant to T20, but also critically dependent on the T20 peptide for its replication. We proposed a mechanistic model that supports this novel feature of drug-dependent viral entry. Briefly, resistance to T20 is caused by the GIA mutation in HR1, which weakens the interaction with both T20 (resistance) and HR2 (gp41 6-helix bundle formation). Reduced HR1-T20 affinity explains the resistance phenotype, but reduced HR1–HR2 affinity negatively impacts Env-mediated fusion and HIV-1 fitness [20,28]. The T20-dependence phenotype is caused by the introduction of the SKY mutation in HR2, which attempts to re-stabilise the HR1–HR2 affinity defect caused by the GIA resistance mutation in HR1. However, the SKY mutation creates a hyper-fusogenic Env-gp41 molecule that prematurely undergoes the conformational switch to a later fusion intermediate or the 6-helix bundle. T20 is able to prevent this premature switch by preserving and earlier pre-fusion conformation, enabling gp41 to undergo the necessary conformational switch at the correct moment in the fusion process. In the present study, we performed forced evolution experiments with the T20-dependent virus in the absence of the T20 peptide with the aim to identify second site compensatory changes, which could provide new mechanistic insights into Env function and the T20-dependence mechanism.

## Results

### The T20-dependent virus evolves to T20-independence

**Figure 1:**
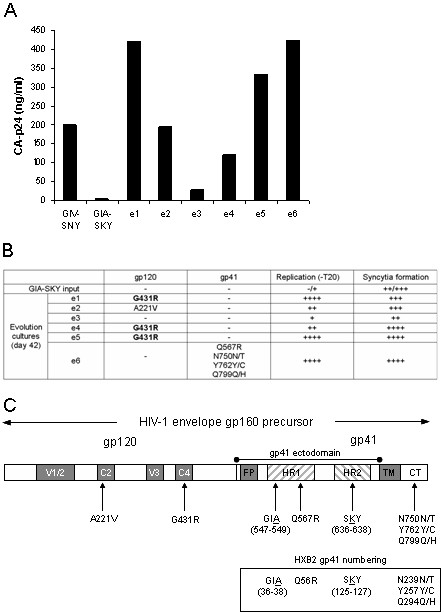
**Evolution of the T20-dependent HIV-1 variant. (A) **Replication of wild-type (GIV-SNY), T20-dependent (GIA-SKY) and HIV-1 variants obtained in evolution cultures in the absence of T20. Bars represent CA-p24 values at day 4 post-infection, which represent the relative differences in replication capacity. Infection of fresh SupT1 cells was performed with an equal amount of the viral stocks obtained at day 42 of evolution. **(B) **Summary of observed mutations, replication capacity and syncytia formation of the six evolution cultures after 42 days of evolution. Replication without T20 and syncytia formation represent relative differences observed in infection experiment displayed in 1A (-, no replication or syncytia; ++++, high replication or all cells involved in syncytia). **(C) **Schematic of mutations in the Env protein. The complete Env gene is shown (not to scale) with the location of the original GIA-SKY mutations and the second site changes selected in the evolution cultures. The G431R change that was selected in multiple cultures is marked in bold. Both gp160 and gp41 HxB2 numbering references are included.

We performed forced evolution experiments with the T20-dependent virus (GIA-SKY) in the absence of the T20 peptide. We previously demonstrated the power of the forced evolution approach in diverse HIV-1 studies [29-31]. Evolution cultures were started by transfection of the GIA-SKY molecular clone into the SupT1 T-cell line. Viral replication was monitored over a 42 day period and virus plus cell samples were taken at several times in the course of the experiment. Efficient viral spread was measured via CA-p24 determination and consequently virus-induced syncytia were observed in 5 of the 6 evolution cultures. The replication capacity of these evolved virus samples was assayed by infection of fresh SupT1 cells with an equal amount of virus. We included the T20-dependent starting virus (GIA-SKY) as well as the wild-type virus (GIV-SNY) as controls (Fig. 1A). Evolution cultures 1, 5 and 6 replicated very efficiently, producing CA-p24 to levels above that of the wild-type and evolution cultures 2 and 4 produced CA-p24 levels similar to the wild-type, confirming the presence of replication competent viral populations that no longer require the T20 peptide. Only evolution culture 3 showed no obvious sign of viral escape.

### Genotypic analysis of the escaped viruses

One may anticipate that the phenotypic reversion would be caused by back-mutation of the SKY sequence in HR2 to the wild-type SNY sequence, which would remove the T20-dependence phenotype. This would produce the GIA single mutant, which is T20-resistant, but no longer dependent on T20 [20]. Although this may seem the most likely outcome, second site compensatory changes elsewhere in the Env glycoprotein would also be a possibility. In order to determine the sequence changes in these phenotypic revertants, we PCR-amplified the entire Env gene from cellular proviral DNA and analysed the sequence for mutational changes (Fig. 1B and 1C). No changes in or directly adjacent to the GIA motif in HR1 or the SKY motif in HR2 were detected. Evolution culture 3, which showed no sign of viral escape, did not reveal any mutational changes within the entire Env gene. All other cultures that yielded an escape viral population showed at least one mutation in the Env gene. Evolution culture 2 showed a single amino acid change A221V in the C2 region of gp120. This amino acid is highly conserved in natural subtype B isolates [52], indicating that it plays an important role in Env function. Evolution culture 6 acquired multiple mutations in gp41 but no changes were seen within gp120. One change was observed in HR1 (Q567R) and 3 partial changes in the cytoplasmic tail (CT), N750N/T, Y762Y/C and Q799Q/H. Of most interest were evolution cultures 1, 4 and 5 that all acquired the same point mutation (G431R; all identical codon changes GGA-to-AGA), with no changes elsewhere in the Env protein. The G431R mutation is positioned in the C4 region of gp120, which plays a critical role in CD4 and co-receptor engagement [32-35]. Amino acid position 431 is highly conserved in natural subtype B isolates [52], which suggests that it plays an important role in Env function. It is striking that the same mutation was selected in 3 independent evolution cultures, suggesting this mutation may play a key role in the phenotypic reversion to T20-independence. We therefore decided to investigate this revertant (GIA-SKY-G431R) in more detail.

### The G431R mutation rescues the T20-dependent virus

**Figure 2:**
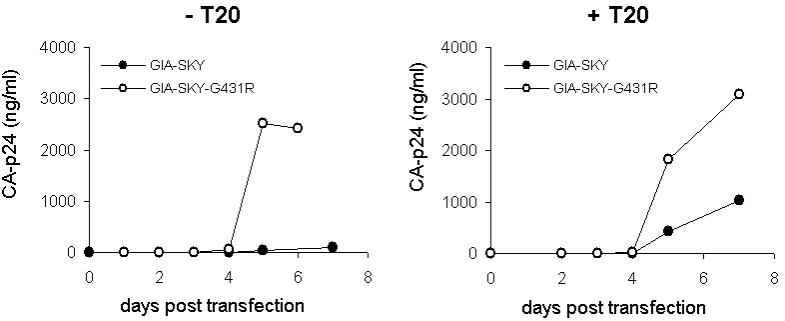
**Replication of the T20-dependent GIA-SKY mutant and the GIA-SKY-G431R revertant virus**. Molecular clones were transfected in SupT1 cells that were cultured with (100 ng/ml) and without T20. Virus replication curves were made over a 7-day period. Closed circles represent the original GIA-SKY mutant virus and open circles the GIA-SKY-G431R revertant. This is a representative experiment, similar results were observed in 3 repeated experiments including results in figure 4, top.

The G431R mutation was introduced into the GIA-SKY molecular clone and tested for its impact on viral replication and T20 resistance/dependence. Viral DNA constructs were transfected into SupT1 cells and cultured in the presence or absence of T20 (Fig. 2). Replication of the GIA-SKY virus was dependent on the T20 peptide as previously reported [20]. The GIA-SKY-G431R revertant was able to replicate in the absence of T20, confirming that the G431R mutation in gp120 is sufficient for the loss of the T20-dependent phenotype. The GIA-SKY-G431R revertant is resistant to T20 because it still contains the crucial T20-resistance mutation GIA.

**Figure 3:**
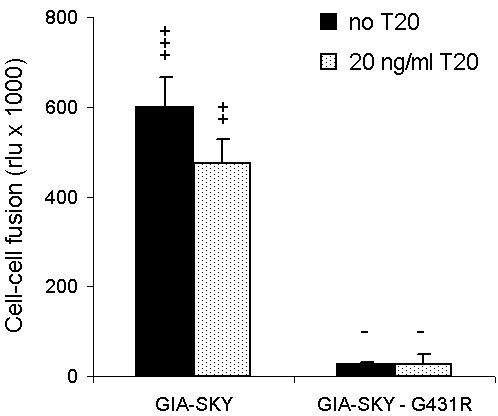
**Cell-cell fusion assay with the GIA-SKY mutant and the GIA-SKY-G431R revertant**. SupT1 cells were transfected with the HIV-1 pLAI constructs indicated below the X-axis. One day later, transfected cells were mixed with SupT1 cells containing a Tat-responsive LTR-luciferase reporter gene construct with (20 ng/ml) or without T20. After 24 hours, formation of syncytia was analysed by light microscopy (-, no syncytia; ++++, all cells involved in syncytia) and quantitated by measurement of luciferase activity in cell extracts.

We next tested the hypothesis that the G431R mutation in the GIA-SKY-G431R revertant represents a compensatory mutation that reduces or controls the structural transition in the GIA-SKY Env protein. We thus propose that the G431R mutation may offer a similar mechanistic check as the T20 peptide and preserve a pre-fusion intermediate necessary for correct gp41 conformational changes. To test this, we performed a cell-cell fusion assay, which specifically measures Env fusion activity (Fig. 3). In this assay, one cell expresses the wild-type or mutant Env protein and the other cell the appropriate receptors (CD4 and CXCR4). Fusion was scored by the formation of syncytia. In addition, we introduced an LTR-luciferase reporter in the acceptor cell that is activated upon cell fusion by Tat protein that is expressed in the donor cell. We measured dramatically reduced fusion activity (syncytia and luciferase counts) for the GIA-SKY-G431R revertant compared to the GIA-SKY mutant (black bars) (Fig. 3). Addition of T20 similarly reduces the fusogenicity of the GIA-SKY mutant (shaded bars), although the effect is more modest than that of the G431R reversion as we only used 20 ng/ml of T20. However, we previously demonstrated that T20 has a dose dependent inhibitory effect in this assay, with higher concentrations significantly blocking cell-cell fusion [20]. This result is consistent with the idea that both the exogenous T20 peptide and the endogenous G431R mutation have a moderating impact on the hyper-fusogenic GIA-SKY Env mutant. They are both able to control or down-regulated the hyper-fusogenicity of the GIA-SKY Env protein, which normally undergoes a premature conformational switch to the 6-helix bundle structure.

### Influence of amino acid 431 on Env function

We decided to investigate the effect of amino acid 431 in more detail. Because the C4 region has been implicated as a contact site for host cell CD4 and co-receptor engagement, amino acid changes in this region may affect the ability of Env to interact with CD4 and/or co-receptor and thereby influence the cell-cell and virus-cell interactions [32-36]. The X-ray structure of the HIV-1 Env protein is consistent with this possibility because amino acid 431 is located within the critical CD4 binding site [37,38]. Alternatively, changes at amino acid 431 may affect the Env-CD4 interaction in a more indirect manner through an effect on the folding of this complex glycoprotein.

**Figure 4:**
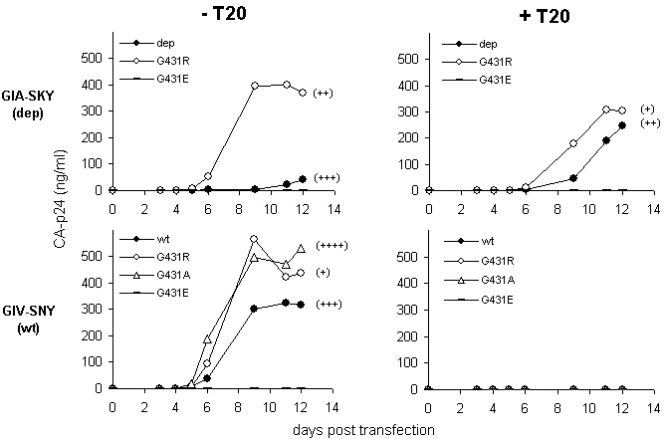
**Replication of G431-mutated viruses**. SupT1 cells were transfected with the T20-dependent (GIA-SKY, top panel) and the wild-type (GIV-SNY, lower panel) with variation at position 431 as indicated. Replication was measured both in the presence (100 ng/ml) and absence of T20. Virus replication curves were made over a 12-day period. Syncytia formation at day 12 was recorded (-, no syncytia; ++++, all cells involved in syncytia). Position 431 variation in the GIA-SKY T20-dependent virus (top) and the wild-type GIV-SNY virus (bottom): wild-type 431G (closed circles), G431R (open circles), G431E (dashes) and G431A (open triangles, in wild-type only).

The T20-independent phenotype is caused by the replacement of a neutral glycine with a positively charged arginine at position 431. We therefore wanted to test what effect the introduction of a negatively charged glutamic acid at this position would have on the GIA-SKY mutant. We constructed the molecular clone GIA-SKY-G431E and tested the ability of this mutant to replicate with and without T20. Transfection of this molecular clone into SupT1 cells resulted in a replication defective virus both with and without T20 (Fig. 4, top). This result demonstrates that amino acid position 431 is critical for Env function and that not any substitution at this position will rescue the GIA-SKY mutant.

We next tested the effect of G431R and G431E on the wild-type virus (GIV-SNY) (Fig. 4, bottom). As expected, replication of the wild-type was strongly inhibited by T20. Introduction of the positive arginine (G431R) resulted in increased replication of the wild-type in the absence of T20. This may be due to the severe syncytia-formation defect that this mutant exhibits in cell culture on SupT1 cells, which allows the cells to survive longer and hence allows the virus to replicate freely and subsequently produce higher quantities of CA-p24 (see discussion for details). Introduction of the negatively charged glutamic acid (G431E) completely abolished viral replication of the wild-type, both with and without T20, as observed in the context of the GIA-SKY virus. However, when an alternative neutral amino acid (alanine which is the most analogous amino acid to the endogenous glycine; G431A) was introduced into the wild-type virus, replication and syncytia formation was re-established. As expected, all C4 mutants in the context of the wild-type sequence were totally sensitive to the T20 peptide (Fig. 4, bottom/right).

### G431R prevents an early gp41 conformational switch by counteracting hyper-fusogenicity

**Figure 5:**
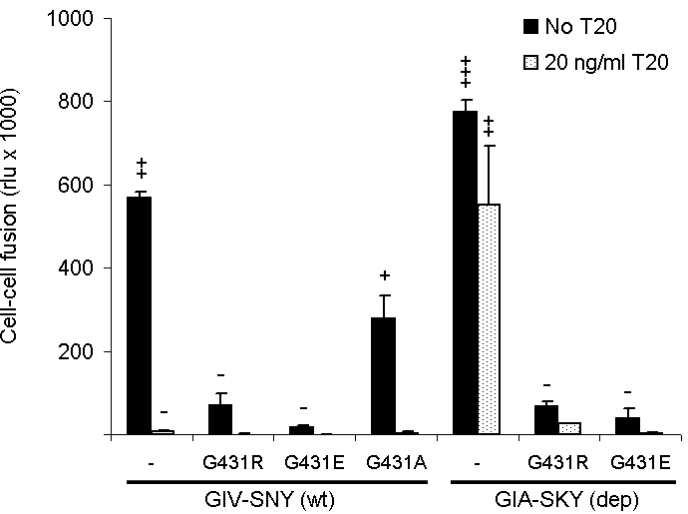
**Cell-cell fusion assay with the G431-mutated Env variants**. Assay of wild-type (GIV-SNY) and T20-dependent (GIA-SKY) viruses with position 431 variation as indicated, in the presence (20 ng/ml) and absence of T20. SupT1 cells were transfected with the mutants indicated below the X-axis. One day later, transfected cells were mixed with SupT1 cells containing a Tat-responsive LTR-luciferase reporter gene construct with (20 ng/ml) or without T20. After 24 hours, formation of syncytia was analysed by light microscopy (-, no syncytia; ++++, all cells involved in syncytia) and quantitated by measurement of luciferase activity in cell extracts.

We further analysed the molecular clones in a cell-cell fusion assay to directly measure their effect on Env function (Fig. 5). As previously reported, GIA-SKY Env is hyper-fusogenic; approximately 1.2–1.5 fold more fusogenic then the wild-type (GIV-SNY) Env and T20 can control this fusion step by inhibiting the wild-type and controlling the hyper-fusogenic activity of the GIA-SKY Env [20]. Indeed, we were able to reproduce this result and show that 20 ng/ml T20 is sufficient to lower the fusogenicity of GIA-SKY back to levels of the wild-type virus. Most importantly, we also measured reduced Env function for the GIA-SKY-G431R revertant and this inhibition was also observed in the presence of T20. G431R had the same effect on Env function in the context of wild-type and this mutant was totally inhibited by T20 as it does not contain the GIA T20-resistance mutation. Introduction of the negatively charged glutamic acid residue completely abolished cell-cell fusion activity of the wild-type virus, consistent with the replication curves of these mutants in Figure 4. The neutral alanine residue in the wild-type context showed intermediate fusion activity, again consistent with the results of replication and syncytia assays shown in Figure 4.

**Figure 6:**
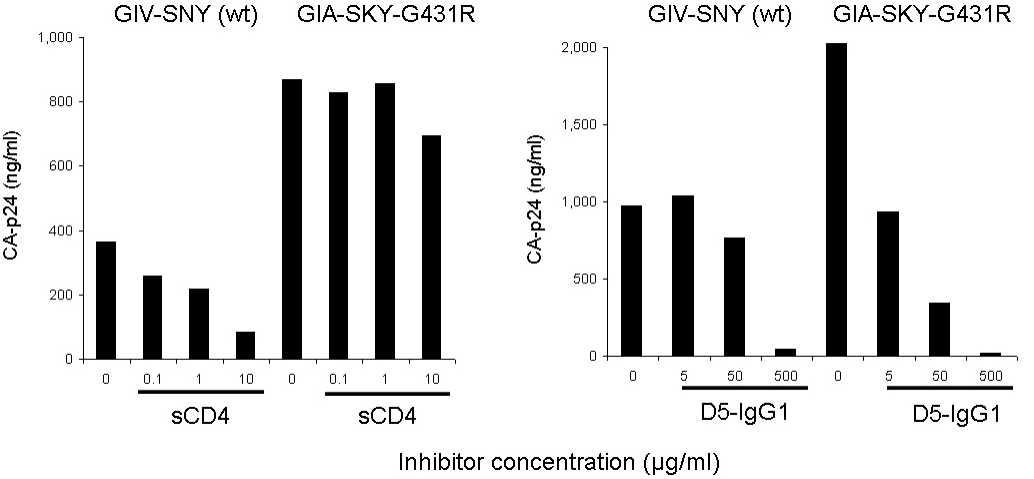
**Sensitivity of wild-type GIV-SNY and GIA-SKY-G431R revertant viruses to sCD4 and D5-IgG1 gp41 antibody**. Bars represent CA-p24 values at day 6 post-transfection, which represent the relative differences in replication capacity in the presence of the sCD4 inhibitor and the D5-IgG1 antibody for the wild-type virus compared to the revertant virus. This is a representative experiment, similar results were observed in 2 independent experiments.

We have shown that GIA-SKY replication in the absence of T20 is facilitated by the introduction of the G431R mutation in the gp120 C4 region. This finding is consistent with our model that GIA-SKY is dead due to an overly aggressive (hyper-fusogenic) Env protein. We propose that G431R negatively affects the Env/CD4 interaction as a means to prevent the abortive premature switch of the 6-helix bundle. To test this, we measured the sensitivity of the wild-type (GIV-SNY) and revertant (GIA-SKY-G431R) viruses to the soluble form of CD4 (sCD4), using a standard virus replication assay to mimic the evolutionary setting (Fig. 6, left panel). sCD4 is able to inhibit virus entry by competitively binding to the Env-gp120 C4 region of HIV-1 before the virus engages the cellular CD4 receptor [39]. The revertant, which produces significantly more CA-p24 compared to the wild-type (see discussion), was less sensitive (more resistant) to sCD4 than the wild-type, indicating that it has reduced CD4 binding affinity. At the highest sCD4 concentration (10 μg/ml), wild-type was inhibited to 23%, whereas the revertant remained at 80% replication capacity. At an intermediate 1 μg/ml sCD4 concentration, wild-type was inhibited to 60% however; this concentration had no inhibitory effect on the revertant.

We further wanted to analyse the gp41 structure of GIA-SKY-G431R revertant and see if this virus is more sensitive to HR1 inhibitors, which would suggest that gp41 is in an "open" or "loose" conformation. For this, we used a gp41 antibody that binds to an open HR1 pre-fusion intermediate. This D5-IgG1 antibody specifically targets the gp41 HR1 region and can bind to a region slightly upstream of the GIA motif [40]. Because the revertant contains the GIA T20-resistant mutation, using T20 is not an option as this revertant is resistant to the peptide (Fig. 2). The revertant was considerably more sensitive (less resistant) to the D5-IgG1 antibody than the wild-type. At the lowest concentration (5 μg/ml), wild-type was unaffected, whereas the revertant was inhibited to 45% replication capacity. At an intermediate 50 μg/ml D5-IgG1 concentration, the revertant was significantly inhibited to 17% replication capacity, whereas the wild-type was inhibited to 78%. This result suggests that the HR1 domain is exposed and more susceptible to the inhibitor in the revertant Env protein. The combined results of the reduced sensitivity to sCD4 and increased sensitivity to D5-IgG1 suggest that the G431R mutation partially restores gp41 function of the Env protein by modulating the Env-CD4 interaction and 6-helix bundle formation.

## Conclusion

In this study, we performed forced evolution experiments with a T20-dependent virus [20] in the absence of T20 with the aim to identify second site compensatory changes, which could provide new mechanistic insights into Env function and the T20-dependence mechanism. Escape variants with improved viral replication were selected in 5 independent evolution cultures. We sequenced the complete HIV-1 Env gene and observed several mutations. Strikingly, 3 evolution cultures contained identical escape variants with the same glycine-to-arginine substitution at position 431 of Env (all identical codon changes GGA-to-AGA). Glycine 431 is located in the CD4 binding region of Env gp120 and is highly conserved among natural HIV-1 isolates, suggesting it plays a key role in CD4 receptor engagement [32-35]. In order to directly test the effect of the G431R mutation, we introduced it back into the original GIA-SKY T20-dependent molecular clone. Replication assays in SupT1 cells confirmed that the G431R mutation is sufficient to restore replication in the absence of T20.

One may anticipate that phenotypic reversion of the T20-dependent GIA-SKY virus would occur via back-mutation of the SKY mutation in HR2 to either SNY or NNY to remove the T20-dependence phenotype. This would produce the GIA single mutant, which is T20-resistant, but not T20-dependent [20]. However, the GIA mutant has diminished replication kinetics, which would place this escape variant at a disadvantage to other escape variants [20,28]. In addition, this evolution route requires a relatively difficult transversion type of mutation (G-to-T), whereas the G431R mutation is made by a simple transition type mutation (G-to-A) [41-43]. Back-mutation of the GIA sequence to GIV is also not an option as the SKY single mutant is a dead virus [20]. This implies that the G431R substitution is selected as an alternative solution to the conformational gp41 defect of the GIA-SKY mutant, which is normally controlled by T20.

We previously reported that T20 is able to control or preserve an early pre-fusion conformation of gp41 so that, after CD4 and co-receptor engagement, gp41 conformational changes can occur and virus/cell fusion and entry can subsequently take place [20]. The G431R mutation could similarly restore gp41 function by preventing the abortive premature switch in gp41. We indeed measured significantly reduced sensitivity to the sCD4 inhibitor (Fig. 6, left panel). Because the C4 region has been implicated as a contact site for CD4 receptor on the host cell surface, these changes may affect the ability of Env to interact with CD4 [32-35]. The X-ray structure of the HIV-1 Env protein is consistent with this possibility because amino acid 431 is located within the critical CD4 binding domain [37,38]. Alternatively, the amino acid 431 change may affect the Env-CD4 interaction in a more indirect manner through an effect on the folding of this complex glycoprotein. To test this, we used a gp41 antibody (D5-IgG1) which targets the HR1 region of the pre-fusion gp41 conformation. For this antibody to have a more potent inhibitory activity, the HR1 region of gp41 would need to be more accessible and hence be in an "open" state, free from bound HR2 [40]. We indeed measured increased sensitivity to the D5-IgG1 antibody (Fig. 6, right panel). The combined results of the reduced sensitivity to sCD4 and increased sensitivity to D5-IgG1 suggest that the G431R mutation partially restores gp41 function of this Env protein by modifying the Env-CD4 interaction and gp41 conformational changes, thus preventing or modulating the premature switch to the 6-helix bundle similar to the effect of the T20 peptide on this mutant.

Interestingly, the G431R mutation has previously been selected in long-term cultures of a translationally impaired HIV-1 mutant [44,45]. Consistent with our current results, reduced syncytia formation and increase CA-p24 production compared to the wild-type virus was also scored. This phenotype allows the infected SupT1 cells to survive longer, thus producing more viral progeny. This loss of fusogenicity via Env-CD4 interaction was measured via a cell-cell fusion assay and provides an obvious advantage in the context of our T20-dependent GIA-SKY mutant which is overly aggressive at engaging target cells, resulting in premature syncytia formation [20]. Beddows *et al*. [17] recently reported a similar finding where a C4 mutant Env showed increased sensitivity to Env antibodies, reduced infectivity and adaptation to SupT1 cells.

In 1987, Kowalski *et al*. [46] demonstrated that insertional mutations in gp41 HR2 region disrupt the gp120-gp41 interaction and suggested that the HR2 region is a "touch point" for gp41-gp120 interactions. More recently, a strong link between the C4 region of gp120 and the gp41-HR2 region (including the T20 sequence) has been proposed by a number of research groups [16-18,46]. Short peptides designed to mimic the C4 region in gp120 have the ability to suppress the T20 inhibitory effect either by preventing T20 from binding to the C4 region in gp120 and/or by modulating gp41 conformational changes via interaction with the HR2 region in gp41. Alam *et al*. [16] found an HR2/T20 peptide-binding site on soluble HIV-1 recombinant gp120. Furthermore, they demonstrated that binding of T20 was induced by sCD4 and anti-gp120 human mAb A32 and was inhibited by the HIV-1 co-receptor-binding site mAb 17b and C4 peptides. Their results strongly suggest a link between the C4 region in gp120 and the gp41-HR2 region. They suggested that a stabilized HR2/Env conjugate may be a possible HIV-1 vaccine candidate with the potential for inducing antibodies against transiently exposed epitopes on HIV-1 Env. It would be interesting to investigate if our GIA-SKY-G431R revertant, which has reduced sensitivity to sCD4 and increased sensitivity to HR1 antibodies, may be such a possible vaccine candidate.

We previously incorporated the T20-dependent phenotype in the doxycycline (dox)-dependent HIV-rtTA virus that was described as a conditional live vaccine strain [47,48]. Our current results indicate that the T20-control may be lost by a single mutation in the Env gene (i.e. G341R). However, we forced this evolutionary escape route by culturing the virus without T20. Furthermore, the dox-control will be used such that the virus is replicating only transiently to induce the immune system and this will restrict evolution and thus avoid unwanted evolutionary paths.

## Materials and methods

### Cell transfection and CA-p24 determination

The SupT1 T-cell line was maintained in RPMI 1640 supplemented with 10% fetal calf serum (FCS), penicillin and streptomycin (both at 100 units/ml) and incubated at 37°C with 5% CO_2_. SupT1 cells were transfected with HIV-1 molecular clones by means of electroporation. Briefly, 5 × 10^6 ^cells were washed in RPMI 1640 containing 20% FCS, mixed with 0.5–5 μg of DNA in 0.4-cm cuvettes, and electroporated at 250 V and 975 μF, followed by resuspension of cells in RPMI 1640 with 10% FCS. The transfected cells were split 24 hours post-transfection and incubated with or without inhibitor or antibody. CA-p24 production was determined from culture supernatant taken at various days post-transfection using a CA-p24 antigen capture enzyme-linked immunosorbant assay as previously described [49].

### Virus evolution in cell culture

For the selection of revertant viruses, SupT1 cells were transfected with 1 μg DNA of the GIA-SKY molecular clone [20]. Transfected cells were split at approximately 16 hours post transfection into 6 separate culture flasks and 0.5 × 10^6 ^fresh SupT1 cells were added in order to start the 6 independent evolution cultures. We initially split 100 μl of cells plus supernatant when required onto uninfected SupT1 cells. When HIV-induced cytopathic effects and increased CA-p24 production were apparent, virus replication was maintained by passage of cell-free culture supernatant onto uninfected SupT1 cells. Initially, we used 100 μl cell-free culture supernatant to infect 5 ml fresh SupT1 cells (approximately 0.5 × 10^6 ^cells), but we gradually used less culture supernatant per passage. Cells and supernatant samples were taken at regular time points and stored at -70°C.

### Proviral DNA isolation, PCR amplification and sequencing

HIV-1 infected cells (1 ml culture) were pelleted by centrifugation at 4000 rpm for 4 min and the supernatant was analysed for CA-p24 contents and stored at -70°C. The cell pellet was lysed in 10 mM Tris-HCl (pH 8.0), 1 mM EDTA, 0.5% Tween 20 and incubated with 500 μg Proteinase K/ml at 56°C for 60 min and heat-inactivated at 95°C for 10 min. Proviral DNA sequences of the entire Env gene were PCR-amplified from solubilized cellular DNA (5 μl) using the Expand High Fidelity PCR System (Roche). Briefly, after incubation for 5 min at 94°C, the reaction mixture was subjected to 35 PCR cycles in a type 9700 DNA thermal cycler (Perkin Elmer Cetus), with each cycle including a denaturation step for 30 sec at 94°C, an annealing step for 30 sec at 60°C and an extension step for 3 min at 68°C. This was followed by a final extension step of 7 min at 68°C. The PCR was performed with 50 ng sense and antisense primers (WS1, 5'-ATAAGCTTAGCAGAAGACA-3', and 3'envMD4, 5'-GCAAAATCCTTTCCAAGCCC-3') in a 50 μl PCR reaction. DNA products were analysed on a 1% agarose gel that was pre-stained with ethidium bromide. PCR products were sequenced directly using the DNA Big Dye Terminator Sequencing Kit (ABI, Foster City, California) and an ABI 377 automated sequencer.

### Construction of LAI molecular clones

The full-length molecular HIV-1 clone pLAI was used to produce wild-type and mutant viruses [50]. We already described the wild-type variant with the GIV-SNY sequence as observed in the patient isolate in place of the GIV-NNY sequence that is present in the LAI molecular clone [20]. The plasmid pRS1, designed to subclone mutant Env genes, was generated as previously described [51]. Mutations were introduced in pRS1 using the Quickchange mutagenesis kit (Stratagene, La Jolla, CA, USA) and the entire Env gene was verified by DNA sequencing. Mutant Env genes in pRS1 were cloned back into pLAI as SalI-BamHI fragments. These included the G431R, G431E and G431A mutations in the C4 region in combination with the gp41 wild-type GIV-SNY or mutant GIA-SKY.

### Cell-cell fusion assay

SupT1 cells (5 × 10^6^) were transfected with 5 μg of the indicated pLAI variants and/or the pcDNA3-Tat expression plasmid as described above. The cells were cultured for approximately 18 hours, spun at 1200 rpm for 10 min and resuspended in fresh media containing saquinavir (1 μM final concentration), with (20 ng/ml) or without T20. Cells were subsequently mixed with SupT1 cells that were transfected the previous day with 5 μg LTR-luciferase reporter plasmid. Cells were cultured for 24 hours, scored for syncytia formation and the cell lysate was assayed for luciferase production (Promega, Madison, WI, USA), which was measured with a luminometer. The output was expressed as relative light units (RLU).
